# Why I tense up when you watch me: Inferior parietal cortex mediates an audience’s influence on motor performance

**DOI:** 10.1038/srep19305

**Published:** 2016-01-20

**Authors:** Michiko Yoshie, Yoko Nagai, Hugo D. Critchley, Neil A. Harrison

**Affiliations:** 1 Human Informatics Research Institute, National Institute of Advanced Industrial Science and Technology (AIST), Tsukuba, 305-8566, Japan; 2 Automotive Human Factors Research Center, AIST, Tsukuba, 305-8566, Japan; 3 Department of Psychiatry, Brighton and Sussex Medical School, Brighton, BN1 9RR, United Kingdom; 4 Department of Life Sciences, Graduate School of Arts and Sciences, The University of Tokyo, Tokyo, 153-8902, Japan; 5 Graduate School of Frontier Biosciences, Osaka University, Osaka, 565-0871, Japan; 6 Sackler Centre for Consciousness Science, University of Sussex, Brighton, United Kingdom

## Abstract

The presence of an evaluative audience can alter skilled motor performance through changes in force output. To investigate how this is mediated within the brain, we emulated real-time social monitoring of participants’ performance of a fine grip task during functional magnetic resonance neuroimaging. We observed an increase in force output during social evaluation that was accompanied by focal reductions in activity within bilateral inferior parietal cortex. Moreover, deactivation of the left inferior parietal cortex predicted both inter- and intra-individual differences in socially-induced change in grip force. Social evaluation also enhanced activation within the posterior superior temporal sulcus, which conveys visual information about others’ actions to the inferior parietal cortex. Interestingly, functional connectivity between these two regions was attenuated by social evaluation. Our data suggest that social evaluation can vary force output through the altered engagement of inferior parietal cortex; a region implicated in sensorimotor integration necessary for object manipulation, and a component of the action-observation network which integrates and facilitates performance of observed actions. Social-evaluative situations may induce high-level representational incoherence between one’s own intentioned action and the perceived intention of others which, by uncoupling the dynamics of sensorimotor facilitation, could ultimately perturbe motor output.

The visible anguish of an athlete whose performance falters at a critical moment conveys the powerful effects that an evaluative audience can exert on motor performance. Empirical evidence suggests that effects of an audience on behaviour can be either facilitative or detrimental, depending on a variety of factors including task demands, performance context, and personality traits^1^,^2^,^3^,^4^. With regard to the type of motor skills, the presence of an audience tends to improve performance of simple (well-learned) tasks such as running and weightlifting^1^,^5^,^6^. However, this socially-induced enhancement of motor output can impair performance of more complex tasks requiring coordination abilities^5^,^7^,^8^. For instance, previous studies^8^,^9^ have shown that the performance quality of skilled pianists deteriorates both technically and artistically, when playing in front of an evaluative audience compared to when playing alone. Further, measurements of electromyographic activity have indicated that persistent low-level muscle tension in the arm and shoulder, induced by the presence of an audience, can lead to increased keystroke force resulting in a loss of fine control of dynamics and temporal fluency within musical performances^8^,^9^,^10^. Although such social effects on motor control are well-documented, the central neural mechanisms through which the presence of observers increases force output have not previously been studied.

To address this, we devised a simple motor task, which was an adaption of earlier pinch- and power-grip tasks^11^,^12^,^13^,^14^ and was designed to capture subtle changes in tonic muscle tension evoked by the presence of an evaluative audience (Fig. 1). We presented two levels of task demand: we asked participants to produce two different levels of grip force, namely 5% or 10% of maximal voluntary contraction (MVC).

The presence or absence of social evaluation was experimentally manipulated by presenting each participant with video footage of two observers who appeared to be closely evaluating the participant’s own task performance in real-time (*observed* condition) or that of another participant (*unobserved* condition). We predicted that the observed condition would elicit a mild level of anxiety in our participants, as in previous studies^8^,^9^. All conditions were implemented within a human functional magnetic resonance imaging (fMRI) environment. By minimising the difference in visual properties of the stimuli used in the two social conditions, we aimed to quantify changes in regional brain activity reflecting effects of social evaluation.

When the presence of evaluative observers influences our motor behaviour, visual information relating to the feelings and intentions of these individuals (e.g., facial expressions, direction of gaze) first needs to be processed in the brain. Previous studies both in monkeys and humans have identified the posterior superior temporal sulcus (pSTS) as a key neural substrate for social perception based on visual cues^15^,^16^,^17^. The pSTS region is known to be activated by actual or implied biological motion (e.g., movements of the eyes, mouth, hands, and body) and also by facial expressions^15^,^18^. Since social evaluation would likely facilitate the processing of these social cues, we predicted that the observed condition would elicit increased activation within the pSTS region.

The inferior parietal cortex (IPC) forms part of the frontoparietal network, and via its anatomical connections with the ventral premotor cortex (PMv) is involved in the sensorimotor transformations necessary for object grasping and manipulation^19^. Given our use of a similar isometric grip task, we hypothesised that effects of social evaluation on motor performance would be mediated via actions on the IPC. Supporting this prediction, during social observation processing of social features within the pSTS region is known to be conveyed to the IPC, which is then capable of generating appropriate motor actions based on the social cues^20^. We thus investigated whether activity within the IPC is modulated in relation to the inter- and intra-individual variability in the effects of social evaluation on force output.

## Results

### Audience effects on anxiety and motor performance

The effectiveness of our social manipulation was evidenced by significantly higher self-rated anxiety in the observed, compared to the unobserved condition (paired *t*_(20)_ = −4.25, *p* < 0.001; Fig. 2a).

As for participants’ motor performance, we observed progressive force decay across time, after the removal of feedback, consistent with previous studies using feedback-occluded isometric force production tasks^11^,^12^,^21^. Magnitude of force decay (force error) was quantified by subtracting the target force from the mean recorded force for each 1-s period of video presentation^11^. Effects were analysed in two-way ANOVAs with factors social condition (observed or unobserved) and target force level (5% or 10% MVC) for each 1-s epoch. Corroborating a previous study that observed greater force decay over time as target force level increased^21^, we observed a significant main effect of target force level on force error for all 15 epochs after the removal of feedback (*p*s < 0.001; Fig. 2b). Force error was consistently larger for the 10% compared to the 5% MVC task. Because of this difference in force error between the two tasks, the presence of an evaluative audience affected motor performance more robustly for the 10% MVC task: social condition × target force level interactions were significant for epochs 15, 14, 13, 10, and 9 (*F*_(1, 18)_ = 8.32, *p* = 0.010; *F*_(1, 18)_ = 7.21, *p* = 0.015; *F*_(1, 18)_ = 4.52, *p* = 0.048; *F*_(1, 18)_ = 5.08, *p* = 0.037; *F*_(1, 18)_ = 4.84, *p* = 0.041, respectively) and marginally significant for epochs 11 and 8 (*F*_(1, 18)_ = 3.73, *p* = 0.069; *F*_(1, 18)_ = 3.67, *p* = 0.071, respectively). Follow-up analyses showed that the difference in force error between the two social conditions was significant for epochs 9–15 for the 10% MVC task (*p*s < 0.05; Fig. 2b), indicating that participants produced significantly stronger grip force in the observed compared to unobserved condition from 8 s after social video onset. For the 5% MVC task, the effect of audience on grip force showed greater individual differences, and did not reach statistical significance at the group level at any time point.

We also calculated correlations between subjective anxiety ratings and the change in force error from the unobserved to observed condition over the last 3-s period of video presentation, where the social condition × target force level interaction was significant. Interestingly, there was a significant positive correlation between the increase in grip force in the 5% MVC task and the Brief Fear of Negative Evaluation Scale^22^ score (*r* = 0.502, *p* = 0.028). This suggests that although the target force level of 5% MVC was too low to produce significant changes in grip force at the group level, individuals high in fear of negative evaluation by others tended to increase their grip force even during the 5% MVC task. Moreover, the increase in force error in the observed condition correlated positively with the increase in state anxiety score in the 10% MVC task (*r* = 0.468, *p* = 0.043).

### Audience effects on functional brain activity

In order to identify the neural substrates underlying effects of social evaluation, we first performed categorical analyses of functional brain activity evoked in the observed compared to unobserved condition. We undertook these analyses separately for the 10% MVC task (Supplementary Table S1) and for the 5% MVC task (Supplementary Table S2). We then tested for brain regions commonly activated or deactivated by the presence of observers in both tasks, which are thought to be related to the effects of social evaluation. This conjunction analysis (Table 1) revealed significantly enhanced activity within the right pSTS (MNI coordinates: [*x*, *y*, *z*] = [50, −31, −2]; *Z* = 4.68) in the observed condition (Fig. 3a). Social evaluation also led to relative deactivations in some brain regions, particularly bilateral IPC ([−39, −58, 43]; *Z* = 4.67; [47, −52, 51]; *Z* = 3.61) encompassing the intraparietal sulcus (IPS) and intermediate areas of the inferior parietal lobule (IPL), as well as bilateral middle frontal gyrus ([−15, 17, 52]; *Z* = 4.70; [27,15,45]; *Z* = 3.88; Fig. 3b).

To further investigate which of these brain regions was especially involved in mediating the effects of social evaluation on force output, we then performed parametric analyses in which we tested for brain regions where activity changed proportionally with socially-induced increases in force production within participants: we first used the unobserved trials to determine the normal level of force decay during the last 1-s period of video presentation, then subtracted this from the force error at each trial to produce a trial-specific index of socially-induced force increase (force increase index). Regression analysis showed that activity within left IPC encompassing intermediate areas of the IPL and IPS ([−44, −48, 46]; *Z* = 3.91) negatively and most robustly correlated with this force increase index (Table 2). No clusters with a significant positive association were found. To exclude effects potentially related to memory-based control of isometric grip force, we tested a second regression model using absolute force values (in % MVC) exerted over the last 1 s of video presentation. This analysis identified correlations with absolute force within left dorsolateral prefrontal cortex ([−39, 48, 4]; *Z* = 3.95), which has previously been implicated in the motor memory processes during isometric force production^12^, and right sensorimotor areas (Table 2). Applying this analysis as an exclusive mask revealed only one region encompassing intermediate areas of the left IPL and IPS ([−44, −46, 45]; *Z* = 3.90) negatively associated with the force increase index (Table 2).

Since the parametric analyses suggested that left IPC disengagement is associated with the motor effects of social evaluation, we further tested if activity within this region also accounted for between-individual differences in motor responses to the audience. To this end, we examined the relationship between the average force increase over the last 3-s period of video presentation and the attenuation of activity within left IPC identified in the categorical analysis by using a linear mixed-effects model with the target force level (5% or 10% MVC) as an independent variable, the force increase as a covariate, and the IPC deactivation as a dependent variable. The results showed that the IPC deactivation was positively and significantly related to the force increase (*F*_(1, 16)_ = 12.37, *p* = 0.003), but not to the target force level (*F*_(1, 27)_ = 0.17, *p* = 0.686), suggesting that individuals with the greatest sensitivity to audience effects on force output also showed the greatest decrease in left IPC activity (Fig. 4a).

Finally, we examined whether social evaluation changed functional connectivity between the two key regions identified above, namely the right pSTS and the left IPC, by performing a psychophysiological interaction (PPI) analysis^23^. The PPI analysis (Table 3) revealed that social evaluation significantly decreased the functional connectivity between the right pSTS and a region encompassing the IPC and the postcentral gyrus ([−41, −24, 48]; *Z* = 4.87) in the left hemisphere. Other regions, including the right IPC ([54, −18, 28]; *Z* = 4.42), also showed reduced coupling with the pSTS. In contrast, social evaluation increased functional connectivity between the right pSTS and bilateral visual areas (Table 3). To investigate whether there was any overlap between the left IPC found in the PPI analysis and that found in the parametric analysis, we searched for brain regions that manifested both decreased connectivity with the pSTS during social evaluation and deactivation in proportion to the force increase index. This conjunction analysis revealed a part of the left IPC encompassing the IPS and intermediate areas of the IPL ([−44, −48, 46]; *Z* = 3.91; Fig. 4b and Table 3).

## Discussion

The present study aimed to investigate the central neural mechanisms underlying effects of social evaluation on fine motor performance. To this end, we asked healthy adults to perform a feedback-occluded isometric grip task at either 5% or 10% MVC, while an evaluative audience was monitoring performance of the participant (observed condition) or that of another individual (unobserved condition). Participants reported significantly higher subjective anxiety in the observed, compared to the unobserved condition, which demonstrated the effectiveness of our social manipulation. Replicating previous findings^11^,^12^,^21^, we observed progressive force decay across time, after the removal of real-time visual feedback of grip force. Interestingly, social evaluation led to the attenuation of this force decay, or the relative increase in grip force during the 10% MVC task. Moreover, participants who experienced the greatest increase in subjective anxiety also showed the greatest increase in grip force in the observed condition. The results are consistent with a previous study showing that the presentation of emotional images attenuated force decay in a similar feedback-occluded isometric grip task at 10% MVC^11^. As for the 5% MVC task, the effect of audience on force output showed greater individual differences: only individuals vulnerable to fear of negative evaluation by others increased their grip force in response to social evalution, and this effect was not significant at the group level. The weaker effect of social evaluation in the 5% MVC task may be partly explained by the fact that the force decay was generally smaller for this condition. Force decay after the removal of visual feedback is suggested to be caused by a decay in information held in short-term visuomotor memory, which in turn reduces the strength of efferent inputs to the motorneuron pool^21^. This decay of visuomotor memory has been shown to become larger as the target force level increases^21^. The social effect on force production was smaller for the 5% MVC task, possibly because this force level was too low for the removal of visual feedback to alter short-term visuomotor memory. All in all, these behavioural results replicate earlier observations that the presence of an evaluative audience can increase muscle activity and the resulting force output^8^,^9^,^10^. Although emotional images^11^,^13^ or monetary rewards^14^ have been reported to elicit a similar enhancement of force output, the present results confirm that the mere presence of others with neutral facial expressions can also alter motor performance. The increased force output under social evaluation can influence motor performance either positively or negatively depending on the type of motor tasks: it would improve the performance of motor tasks that mainly demand power or speed (e.g., weightlifting, running)^1^,^5^,^6^, but would impair the performance of tasks demanding fine motor coordination (e.g., figure skating, piano playing)^3^,^5^,^8^,^9^.

As hypothesised, our fMRI data related effects of social evaluation to activation within the right pSTS. Both electrophysiological recordings in monkeys and neuroimaging studies in humans show that the pSTS is sensitive to social cues involving actual or implied biological motion and also to the direction of others’ gaze and/or attention^15^,^16^,^17^. The region is further implicated in mentalization and theory of mind processes (i.e., knowing what another person may be thinking)^24^,^25^. The right pSTS is especially involved in the recognition of facial emotion^18^. Therefore, the right pSTS activation during the observed condition likely reflects processing of both the direction of the two judges’ attention and their potential emotional responses to participants’ performance.

On the other hand, the bilateral IPC was deactivated by the presence of an evaluative audience. The parametric analyses demonstrate that activity within the left IPC in particular was modulated by both within- and between-individual variability in the effects of social evaluation on force output. The deactivation within the IPC contralateral to the gripping hand reflects the changes in force output due to social evaluation. In monkeys, connections between the IPC and the PMv form a major part of the frontoparietal network involved in visuomotor transformations necessary for object grasping and manipulation^19^. Thus the IPC integrates somatosensory inputs from the contralateral body, as evidenced by the effect of lesions to this region which may cause alien hand syndrome in the contralateral limb^26^. Moreover, the left IPL is specifically involved in the integration of kinesthetic information of hand movement and tactile information from a touched object during hand-object interactive movements^27^. This integration is crucial for the present isometric grip task. Supporting this idea, an fMRI study employing an isometric precision-grip task with the right hand revealed that the left IPC was more strongly activated during gentle grip (~1.7% MVC) than normal (~2.8% MVC) or firm (~5.5% MVC) grip^28^. Taken together, the IPC subserves the sensorimotor integration required for the fine control of isometric force production. The IPC deactivation induced by social evaluation may thus correspond to an attenuation of sensorimotor representations, ultimately leading to heightened tonic muscle tension, engendering a loss of movement fluency^3^,^8^.

The present findings also provide further support for the view that the left IPC plays an important role in motor memory. Neuroimaging studies in humans suggest that the left IPC is involved in the storage of acquired skill^29^. Clinically, acquired apraxia, which manifests itself as the inability to perform previously learned actions, is often a consequence of damage to the left IPC^30^. Observations both in music and sport indicate that social evaluation appears to induce a temporary regression to an earlier stage of motor learning, characterised by tonic muscle tension and the loss of fluency in movements^8^,^10^,^31^. The deactivation in the left IPC evoked by social evalution may therefore explain such altered motor performance and be seen as transient functional apraxia.

It is also noteworthy that the pSTS-IPC-PMv circuit overlaps with the descriptions of the mirror neuron system^20^,^32^,^33^ or action-observation network (AON)^34^,^35^. When primates observe and/or imitate others’ actions, the visual representation of the movements within the pSTS is relayed first to the IPL, and then to the PMv and adjacent posterior IFG^20^,^34^,^35^,^36^. This pattern of neural activity helps primates understand others’ actions from a first-person perspective^37^. The same network is further engaged in the recognition of facial emotional expressions and in inferring the underlying mental state of others^20^,^38^. Therefore, our observation that the IPC was *deactivated* during the expression of motor audience effects indicates that social evaluation disengages a network that monitors states of both self and others. This is supported by recent studies in monkeys demonstrating that a significant portion of mirror neurons are suppressed during action observation^39^,^40^,^41^.

The human AON is optimally engaged when movements of self and others are identical (imitative action)^20^,^34^ or complementary (joint action)^42^,^43^. However, there is a paucity of research investigating how the AON responds to situations where movements or intentions of self and others are incoherent. Using a typical example of such an incoherent social situation (social evaluataion), we identified a novel pattern of response within the AON, namely the pSTS activation and the IPC deactivation, accompanied by the attenuation of functional connectivity between the two regions. This uncoupling within the AON may be explained in part by predictive coding within this system^34^,^41^. When one is engaged in movements that are independent from those of others, the brain must suppress somatosensory prediction errors of the AON that would otherwise cause one to automatically mimic others^44^, a function that has been proposed to be supported by suppression mirror neurons^41^. This functional modulation of the AON is likely to be maximal when the perceived intentions or actions of others are different from one’s own, and may even obstruct one’s successful motor performance. The IPC deactivation and its reduced coupling of activity with the pSTS may serve as a mechanism that helps performers to detach their attention from the visual stimuli of an evaluative audience and to maintain their motor performance. Together, we propose that incoherence between self and others disrupts the typical facilitatory functioning of the AON, altering skilled motor control. The implications from the present study lead to a better understanding not only of the neural mechanisms underlying effects of social evaluation, but importantly how our brain, within a broader network, integrates and manages social influences on self-initiated behaviour.

## Methods

### Participants

Participants were 21 healthy individuals (11 females; mean age ± SD = 25.5 ± 6.1 years). All were right handed, with a mean (±SD) Edinburgh Handedness Inventory^45^ score of 87.0 (±13.4). All had normal or corrected-to-normal vision, no structural brain abnormality, and no past neurological or psychiatric history. All denied use of medication or recreational drugs. Participants were recruited via the University of Sussex psychology subject database and the Brighton Gumtree website. All participants gave written informed consent prior to the experiment. The study was approved by the Brighton and Sussex Medical School Ethics Committee, and was carried out in accordance with the approved guidelines.

### Experimental procedure

#### Grip force measurement and feedback

During scanning participants held a purpose-built pressure sensor between the fingers and thumb of their right hand, while resting the right arm over the abdomen. Their left arm was extended down the left side of the body and held relaxed. The pressure sensor was made of an air-filled bottle connected via incompressible plastic tubing to a pressure transducer (Keller PR-21; Keller, Winterthur, Switzerland) capable of converting air pressure into voltage. The pressure device was calibrated and generated a differential voltage signal that was linear across the range 0 to 1 bar, fully covering the pressure ranges produced by participants. Voltage signals were passed to a Cambridge Electronic Design Power1401 data acquisition interface and digitally recorded at 1 kHz on a dedicated computer running Spike2 software. In order to provide participants with real-time visual feedback on their exerted force, a customised Spike2 script averaged the voltage signals every 200 ms and sent this via a serial port to the stimulus presentation computer. Presentation of visual stimuli was programmed with Cogent 2000 (http://www.vislab.ucl.ac.uk/cogent_2000.php) within Matlab 7.8 (MathWorks, Natick, MA, USA), and displayed on a projector screen visible via a mirror positioned on the head coil.

#### Force calibration and practice sessions

**Figure 1:**
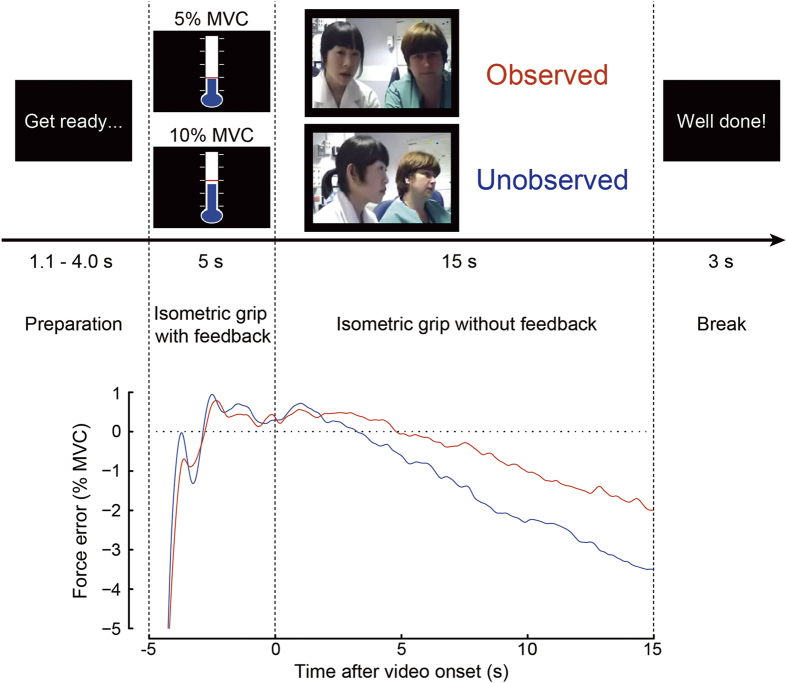
Experimental task. Participants first viewed a thermometer and adjusted their grip force (blue liquid) to match the target force (5% or 10% of their maximal voluntary contraction or MVC, red line) for 5 s. They then tried to maintain the target force for 15 s while viewing a video of two judges apparently monitoring either their own (observed condition) or another participant’s performance (unobserved condition). The lower panel shows a typical example of participants’ performance during the 10% MVC task. Progressive force decay was quantified by subtracting target from actual force (force error). Participants produced stronger grip force in the observed (red) compared to unobserved (blue) condition.

**Figure 2:**
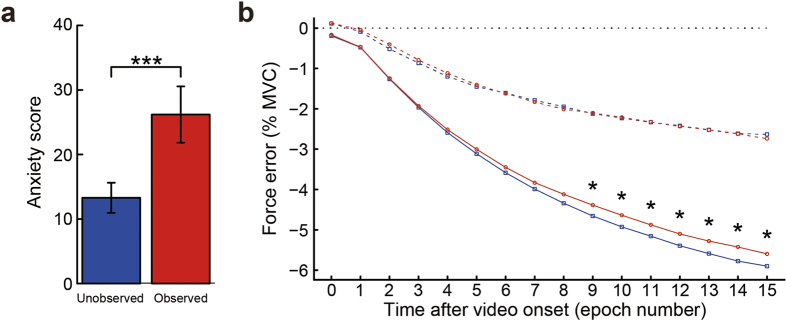
Behavioural results. (**a**) Mean self-rated anxiety score showing a significant increase in anxiety in the observed condition. (**b**) Mean force error. Force error data for each 1-s period of social video presentation were averaged across trials and participants for each condition. During the 10% MVC task isometric grip force was significantly increased in the observed (red solid line) compared to unobserved condition (blue solid line). However, during the 5% MVC task force level did not significantly differ between the observed (red dashed line) and unobserved (blue dashed line) conditions at the group level. Data are represented as mean ± SEM. ***p < 0.001, *p < 0.05.

**Figure 3:**
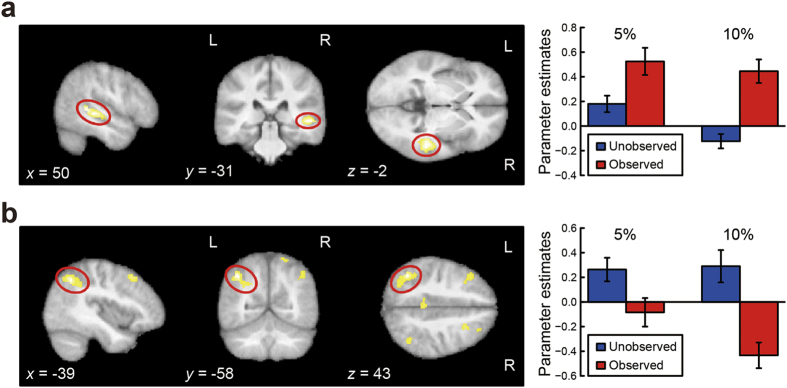
fMRI results of categorical analyses. (**a**) Right posterior superior temporal sulcus (pSTS) showed activation in the observed condition (Conjunction observed >unobserved in both 10% and 5% MVC tasks). (**b**) Left inferior parietal cortex (IPC) showed deactivation in the observed condition (Conjunction observed <unobserved in both 10% and 5% MVC tasks). The bar graphs show parameter estimates extracted from a 4-mm radius sphere placed around the group peak within the circled regions for each condition. Data are represented as mean ± SEM. Clusters are illustrated at *p* < 0.001.

**Figure 4:**
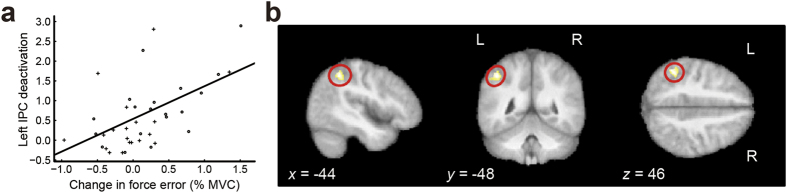
fMRI results of parametric analyses. (**a**) Individual changes in force error were positively and significantly related to the decrease in parameter estimates of the left IPC cluster from the unobserved to observed condition. The pluses denote the 5% MVC task and the circles the 10% MVC task. (**b**) The left IPC showed both deactivation in proportion to the socially-induced increase in force production and decreased functional connectivity with the right pSTS during social evaluation. Clusters are illustrated at *p* < 0.001.

Before starting the main experiment each participant was asked to lightly hold the pressure sensor for 10 s to calibrate the baseline. MVC was obtained by asking the participant to squeeze the bottle as hard as possible for 5 s. MVC was determined as the maximum value obtained during this period. During the task, the force exerted on the pressure sensor was displayed on the projector screen as a blue fluid level moving up and down within a ‘thermometer’ display marked with five scale points, corresponding to baseline, 5, 10, 15, and 20% MVC values, respectively (Fig. 1).

Each participant performed two practice trials to familiarise himself/herself with the stimulus presentation and isometric grip manipulation. In the first trial, the participant was required to adjust and maintain their force level for 20 s to match a red target line positioned at 10% MVC. In the second trial, the participant was asked to match the target force (10% MVC) for the initial 5 s. Visual feedback was then occluded and the participant was instructed to maintain the same level of force over the following 15 s without feedback.

#### Experimental tasks

Each participant performed two sessions of isometric grip task with or without social evaluation, while undergoing fMRI. Each session lasted ~19 min and consisted of 40 trials, including 10 repetitions of each of the four trial types. The four trial types corresponded to a 2 × 2 factorial design: two social conditions (observed or unobserved) × two target force levels (5% or 10% MVC). A schematic diagram of the isometric grip task is shown in Fig. 1. Random jitter (1.1–4.0 s) was inserted prior to each trial to ensure better sampling of the hemodynamic response and maintain participant engagement.

At trial onset (marked by the appearance of the thermometer), the participant started to squeeze the pressure sensor and adjusted the level of force production to match the observed target force. Following this initial 5-s period, the picture of thermometer was replaced by 15-s video footage showing the faces of two experimenters sitting in the MRI control room. Although the participant was led to believe that the video was in real time, the footage was actually pre-recorded^46^. Before starting the experimental trials, each participant was told that another participant would be simultaneously performing the same task in the adjacent scanner. On 50% of trials, the video appeared to show the two observers monitoring and discussing the actual participant’s performance (*observed* condition), and on the other 50% the observers appeared to be monitoring another participant’s performance (*unobserved* control condition). We produced 20 different videos for the observed and unobserved conditions for each of the four possible pairs of experimenters associated with the study. The videos corresponding to the pair of experimenters present on the study day were used. Before scanning the two experimenters greeted the participant, wearing the same clothes as worn in the video footage to ensure that the participant believed that he/she was looking at the experimenters’ faces in real time through a web camera. A fixed 3-s break was inserted after each trial. The two target force levels and two social conditions were randomly distributed over the session and the videos were presented in a randomised order.

### Behavioural data analyses

#### Grip task performance

All behavioural data were analysed in Matlab 7.8 using purpose-written routines. Air pressure data sampled at 1 kHz were low-pass filtered at 2 Hz and normalised relative to MVC (% MVC). The normalised force data were segmented into epochs of 16 s (i.e., from 1 s prior to the onset of video to the end of video). The degree of force decay was quantified by subtracting the target force from the mean recorded force during each 1-s period of social video presentation (force error)^11^. Due to technical problems (i.e., air leakage from the pressure grip), we could calibrate the force data for 19 of the 21 participants. Therefore, the data from these 19 participants were used in the analyses involving force error.

We first conducted a repeated-measures ANOVA on the force error data with three repeated factors: social condition (2 levels: observed or unobserved), target force level (2 levels: 5% or 10% MVC), and time (16 levels: epochs 0–15). Because the social × force × time interaction effect was found to be highly significant (*F*_(15, 270)_ = 3.96, *p* < 0.0001), we subsequently performed separate two-way ANOVAs with two repeated factors (i.e., social condition and target force level) for each 1-s epoch.

#### Anxiety self-ratings

After the fMRI experiment, participants retrospectively rated their level of state anxiety while viewing the social video footage using a visual-analogue scale (VAS), which has been shown to provide a quick, reliable, and relatively sensitive measure of emotional state^47^. The 100-mm VAS ranged from 0 (*not anxious at all* on the left end) to 100 (*extremely anxious* on the right end). We asked participants to place a vertical line bisecting the 100-mm VAS to indicate their perceived level of anxiety during the observed and unobserved conditions. Paired *t*-test was then used to assess the change in VAS score from the unobserved to observed condition. We also asked participants to complete the Brief Fear of Negative Evaluation Scale^22^, which consisted of 12 items concerning fear of negative evaluation by others (e.g., Sometimes I think I am too concerned with what other people think of me.). Participants rated each item on a scale ranging from 1 (*not at all characteristic of me*) to 5 (*extremely characteristic of me*). The relationships between change in force error from the unobserved to observed condition and anxiety ratings were examined using Pearson’s product-moment correlation coefficients.

### Scanning and imaging data analyses

#### MRI acquisition and image preprocessing

Whole brain fMRI data were acquired on a 1.5 T Siemens Magnetom Avanto scanner (Siemens, Erlangen, Germany) at the Clinical Imaging Sciences Centre, Brighton and Sussex Medical School using a 12-channel head coil. We obtained T2*-weighted echo-planar images (EPI) with blood-oxygen level-dependent (BOLD) contrast, each comprising a full volume of 33 slices (slice thickness = 3.0 mm, inter-slice gap = 0.75 mm, in-plane resolution = 3.0 × 3.0 mm, TR = 3300 ms, TE = 50 ms). An average of 345 volume images were acquired for each participant per session (~19 min), with the first five volumes of each session discarded to allow for T1 equilibration effects. During the break between the two sessions, T1-weighted structural images were acquired. At the end of the experiment, gradient field maps were also acquired for each participant to enable subsequent unwarping of functional images with regard to the B0 field.

fMRI data were preprocessed using Statistical Parametric Mapping 8 (SPM8; http://www.fil.ion.ucl.ac.uk/spm) running in Matlab 7.8. Structural images were co-registered with the mean EPI, segmented and normalised to a standard T1 template, and averaged across all participants to allow group-level anatomical localization. All fMRI results were overlaid onto this average anatomical image. Functional images were realigned to the first image of each session, unwarped, and spatially normalised using parameters from segmentation of the T1 structural images, and spatially smoothed with a Gaussian kernel of 8 mm full width half maximum (FWHM).

#### fMRI data analysis

Statistical analyses were performed using block designs on SPM8. The 5-s periods of isometric grip with visual feedback of force (thermometer phase) and the 15-s periods of social video presentation (video phase) were modelled as blocks of corresponding duration. Participant-specific realignment parameters were modelled as covariates of no interest to correct for motion artefacts. Data were corrected for the effects of serial auto-correlations and high-pass filtered (cutoff = 128 s) to remove low-frequency drifts.

In the first categorical analysis, 6 separate regressors were created for the 2 stimuli conditions of the thermometer phase (2 target force levels) and the 4 stimuli conditions of the video phase (2 social conditions × 2 target force levels) and convolved with a canonical hemodynamic response function to model corresponding changes in BOLD contrast signal. The general linear model was used to generate parameter estimates of block-related activity for each voxel, for each trial type and participant. Group-level random-effect analyses were performed using repeated-measures ANOVAs on the contrast images obtained for each of the 2 × 2 factor combinations of the video phase for each participant. We first examined BOLD responses to the presence of observers (observed >unobserved and observed <unobserved) separately for the 5% and 10% MVC tasks. To elucidate neural mechanisms commonly underlying the effecs of social evaluation in both tasks, we then conducted a conjunction analysis^48^ to identify the intersection of the regions activated or deactivated to the 5% and 10% MVC contrasts. To this end, we applied inclusive masking of the 5% MVC contrasts (*p* < 0.05, uncorrected) to the 10% MVC contrasts.

Next, to examine more in detail brain activity associated with motor effects of social evaluation, we performed independent parametric analyses by using 2 separate models. In both models, 2 separate regressors were created for all the conditions of the thermometer phase and all the conditions of the video phase. For the thermometer phase, the maximum force produced by each participant during this 5-s period was included as a parametric modulator. In the first model, we computed the parametric modulator for the video phase as follows: we first determined the normal level of force decay (normal force error) separately for the 5% and 10% MVC tasks for each participant by averaging the force error for the last 1-s period of video phase across all 10 unobserved control trials of each session. We then subtracted the normal force error of the corresponding force level (5% or 10% MVC) from the actual force error of the last 1-s period of video phase for each trial for each participant (force increase index). This force increase index allowed us to test for brain regions whose activity proportionally changed with socially-induced increases in isometric grip force on a trial-by-trial basis. Linear contrasts of regression coefficients were computed at the individual level and taken to a group-level random-effect analysis (one-sample *t*-test) to investigate brain activity correlating with this force increase index. These linear contrasts might also contain clusters that simply correlate with the level of exerted force. To exclude such brain regions, we produced a second model where we included absolute force value (in % MVC) of the last 1-s period of video phase as a parametric modulator. The brain regions emerging from the second model should be related to the memory-based control of isometric grip force in general. Therefore, to isolate brain regions involved in motor audience effects, we removed voxels with significant correlation (*p* < 0.005, uncorrected) in the second model from those in the first model with exclusive masking.

Since the parametric analyses indicated that the left IPC played a key role in mediating the social effect on isometric force production, we further tested if the left IPC activity also accounted for individual differences in motor responses to social evaluation. To this end, we computed differences in parameter estimates between the observed and unobserved conditions for the 5% and 10% MVC tasks separately, from a 4-mm radius sphere placed around the group peak within the left IPC for each participant. We then investigated whether there is a significant relationship between the changes in force error over the last 3-s period of video presentation and those in IPC activity from the unobserved to observed condition by using a repeated-measures linear mixed-effects model with the target force level (5% or 10% MVC) as an independent variable, the force increase as a covariate, and the IPC deactivation as a dependent variable. A preliminary analysis suggested that the relationship between the covariate and the dependent variable did not significantly differ as a function of the independent variable (*F*_(1, 31)_ = 0.18, *p* = 0.672). Since the homogeneity-of-slopes assumption was met, we subsequently performed the linear mixed-effects analysis.

We also examined whether social evaluation changed functional connectivity between the two key regions identified in the categorical and parametric analyses, namely the right pSTS and the left IPC, by performing a PPI analysis^23^. We first extracted the time series of data from a 4-mm radius sphere placed around the peak within the right pSTS for each participant. The interaction term was computed by multiplying a task vector reflecting the social condition (1 for the observed and −1 for the unobserved condition; convolved with a canonical hemodynamic response function before multiplication) with the source regressor. The general linear model was fitted to the PPI regressor, the source regressor, and the task vector for each participant. The group-level random-effect analysis was then performed using a one-sample *t*-test on the contrast images obtained for the PPI regressor. Finally, we examined whether there was any overlap between the left IPC found in the PPI analysis and that found in the parametric analysis. We tested for brain regions that decreased connectivity with pSTS in the observed condition and also showed deactivation in proportion to the force increase index by applying inclusive masking of the PPI contrasts (*p* < 0.05, uncorrected) to the contrasts derived from the parametric analysis.

To determine a cluster extent threshold that corresponds to a threshold of *p* < 0.05 after correction for multiple comparisons, we conducted 1,000 simulations of whole-brain fMRI activation assuming a type I error voxel activation probability of 0.001^49^,^50^. Based on these simulations, we reported only clusters containing a minimum of 120 resampled voxels.

## Additional Information

**How to cite this article**: Yoshie, M. *et al.* Why I tense up when you watch me: Inferior parietal cortex mediates an audience’s influence on motor performance. *Sci. Rep.* **6**, 19305; doi: 10.1038/srep19305 (2016).

## Supplementary Material

Supplementary Information

## Figures and Tables

**Table 1:** Regions commonly activated or deactivated by social evaluation in the 10% and 5% MVC tasks.

Brain region	Side	Number ofvoxels	MNI coordinates(mm)			*t*value	*Z*score	*p*FWE
Activation (observed>unobserved)								
Superior temporal sulcus	R	802	50	−31	−2	5.04	4.68	0.003
Deactivation (observed < unobserved)								
Middle/Superior frontal gyrus	L	1124	−15	17	52	5.06	4.70	<0.001
Intraparietal sulcus (hIP1, 2, 3)/Inferior parietal lobule (PGp)	L	840	−39	−58	43	5.02	4.67	0.002
Postcentral gyrus (BA2, 1)/Superior parietal lobule (7PC)	R	534	29	−46	67	4.49	4.22	0.020
Middle frontal gyrus	R	401	27	15	45	4.08	3.88	0.060
Inferior parietal lobule (PFm, PGa, PF)/Intraparietal sulcus (hIP1)	R	317	47	−52	51	3.78	3.61	0.125
Superior occipital gyrus	R	233	26	−75	35	3.79	3.62	0.267
Calcarine gyrus (V1, V2, V3)	R	229	9	−87	4	3.90	3.72	0.277
Cuneus (V3, V2)	R	156	6	−85	27	3.94	3.76	0.523
Superior parietal lobule (5Ci, 5M)	L	148	−15	−37	39	4.63	4.35	0.558

*Notes.* BA, Brodmann area; L, left; R, right. Only clusters containing a minimum of 120 resampled voxels at an uncorrected *p* < 0.001 are reported. This combination of voxel-level and extent threshold is computed to be equivalent to a threshold of *p* < 0.05 after correction for multiple comparisons (see Methods). The rightmost column shows conventional application of family-wise error (FWE) corrected *p* values at the cluster level.

**Table 2:** Regions with significant deactivation correlating with parametric modulators.

Brain region	Side	Number ofvoxels	MNI coordinates(mm)			*t*value	*Z*score	*p*FWE
Force increase index								
Inferior parietal lobule (PFm, PGa, PF)/Intraparietal sulcus (hIP2)	L	795	−44	−48	46	5.01	3.91	<0.001
Precentral gyrus (BA4, 6)/Postcentral gyrus (BA3)	R	244	30	−33	51	4.95	3.88	0.101
Absolute force level								
Middle frontal gyrus	L	252	−39	48	4	5.07	3.95	0.052
Precentral gyrus (BA4)/Postcentral gyrus (BA3)	R	202	35	−30	51	5.40	4.11	0.116
Precentral gyrus (BA6)	R	198	24	−13	73	6.80	4.73	0.123
Paracentral lobule (BA4, 6)	R	188	3	−31	60	4.95	3.88	0.145
Inferior parietal lobule (PGp, PGa)	L	170	−42	−69	25	5.25	4.04	0.196
Force increase index-Absolute force level								
Inferior parietal lobule (PFm, PF)/Intraparietal sulcus (hIP2, 1)	L	484	−44	−46	45	4.99	3.90	0.005

*Notes.* BA, Brodmann area; L, left; R, right. Only clusters containing a minimum of 120 resampled voxels at an uncorrected *p* < 0.001 are reported. This combination of voxel-level and extent threshold is computed to be equivalent to a threshold of *p* < 0.05 after correction for multiple comparisons (see Methods). The rightmost column shows conventional application of family-wise error (FWE) corrected *p* values at the cluster level.

**Table 3:** Regions with significant changes in functional connectivity with pSTS.

Brain region	Side	Number ofvoxels	MNI coordinates(mm)			*t*value	*Z*score	*p*FWE
Increase in connectivity (observed > unobserved)								
Superior occipital gyrus/Lingual gyrus/Calcarine gyrus (V2, V3, V1, V4)	L	1625	−9	−97	12	5.75	4.37	<0.001
Middle temporal gyrus/Inferior occipital gyrus (V4, V5/MT)	R	740	50	−73	3	5.76	4.37	<0.001
Middle occipitalgyrus (V5/MT)	L	139	−48	−76	7	4.74	3.83	0.452
Decrease in connectivity (observed < unobserved)								
Postcentral gyrus (BA3, 4)/Inferior parietal lobule (PFt, PFop)	L	2283	−41	−24	48	6.88	4.87	<0.001
Insula lobe/Rolandic operculum	L	622	−39	0	10	5.43	4.21	0.002
Posterior medial frontal gyrus	L	502	−8	9	42	5.05	4.01	0.006
Inferior parietal lobule (PFt, PFop)	R	401	54	−18	28	5.85	4.42	0.018
Superior frontal gyrus	R	311	23	2	58	4.99	3.98	0.052
Parahippocampal gyrus	L	120	−15	−27	−15	4.93	3.94	0.562
Conjunction (Decrease in connectivity with pSTS & Correlation with force increase index)								
Intraparietal sulcus (hIP2, 3, 1)/Inferior parietal lobule (PF, PFt)	L	257	−44	−48	46	5.01	3.91	0.084

*Notes.* BA, Brodmann area; L, left; R, right. Only clusters containing a minimum of 120 resampled voxels at an uncorrected *p* < 0.001 are reported. This combination of voxel-level and extent threshold is computed to be equivalent to a threshold of *p* < 0.05 after correction for multiple comparisons (see Methods). The rightmost column shows conventional application of family-wise error (FWE) corrected *p* values at the cluster level.
