# Supplementary material for: Why I tense up when you watch me: Inferior parietal cortex mediates an audience’s influence on motor performance
Source: Sci Rep. 2016 Jan 20;6:19305. doi: 10.1038/srep19305 (PMC4726313; doi:10.1038/srep19305)
Supplement: Supplementary Information [file srep19305-s1.pdf]

Supplementary Information

Why I tense up when you watch me: Inferior parietal cortex mediates an audience's influence on motor performance

Michiko Yoshie, Yoko Nagai, Hugo D. Critchley, Neil A. Harrison

Supplementary Table S1. Regions activated or deactivated by social evaluation in the 10% MVC task.

Brain region	Side	Number of voxels	MNI coordinates (mm)			*t* value	*Z* score	*p*FWE
**10% observed > 10% unobserved**								
Superior temporal sulcus	R	958	50	-31	-2	5.04	4.68	< 0.001
Superior temporal sulcus	L	196	-50	-6	-15	4.26	4.03	0.372
Superior temporal sulcus	L	122	-54	-22	-3	4.50	4.24	0.679
**10% observed < 10% unobserved**								
Middle/Superior frontal gyrus	L	1194	-15	17	52	5.06	4.70	< 0.001
Intraparietal sulcus (hIP1, 3, 2) / Inferior parietal lobule (PGp)	L	1037	-39	-58	43	5.02	4.67	< 0.001
Postcentral gyrus (BA2, 1) / Superior parietal lobule (7PC, 5L)	R	563	29	-46	67	4.49	4.22	0.016
Middle frontal gyrus	R	448	27	15	45	4.08	3.88	0.040
Inferior parietal lobule (PFm, PGa, PF) / Intraparietal sulcus (hIP1)	R	425	47	-51	50	3.78	3.62	0.049
Superior parietal lobule (5Ci, 5M)	L	312	-15	-33	36	5.12	4.75	0.131
Superior occipital gyrus	R	233	26	-75	35	3.79	3.62	0.267
Calcarine gyrus (V1, V2)	R	229	9	-87	4	3.90	3.72	0.277
Inferior parietal lobule (PFt, PF, PFop)	L	189	-57	-28	36	4.31	4.08	0.396
Inferior/Middle frontal gyrus	L	162	-36	44	12	3.70	3.55	0.498
Cuneus (V3, V2)	R	156	6	-85	27	3.94	3.76	0.523
Inferior frontal gyrus (BA44)	L	144	-45	0	22	4.73	4.43	0.576

Notes. BA, Brodmann area; L, left; R, right. Only clusters containing a minimum of 120 resampled voxels at an uncorrected $p < 0.001$ are reported. This combination of voxel-level and extent threshold is computed to be equivalent to a threshold of $p < 0.05$ after correction for multiple comparisons (see Methods). The rightmost column shows conventional application of family-wise error (FWE) corrected *p* values at the cluster level.

Supplementary Table S2. Regions activated or deactivated by social evaluation in the 5% MVC task.

Brain region	Side	Number of voxels	MNI coordinates (mm)			*t* value	*Z* score	*p*FWE
**5% observed > 5% unobserved**								
Superior temporal sulcus	R	189	53	-24	-5	4.10	3.89	0.396
Lingual gyrus	L	130	-9	-72	-6	4.44	4.19	0.641
**5% observed < 5% unobserved**								
Lingual/Calcarine gyrus (V2, V3, V1, V4)	R	3478	9	-76	-5	4.61	4.33	< 0.001
Parahippocampal gyrus	L	472	-20	-39	-8	5.34	4.92	0.033
Superior parietal loble (7A)	L	305	-29	-78	36	3.75	3.59	0.140
Intraparietal sulcus (hIP1, 3)	L	271	-35	-40	37	4.15	3.93	0.190
Parahippocampal gyrus	R	266	27	-37	-12	4.41	4.16	0.198
Middle/Superior frontal gyrus	L	247	-21	11	52	3.92	3.74	0.236
Middle frontal gyrus	L	188	-33	18	42	4.13	3.92	0.399
Superior frontal gyrus	L	173	-17	71	12	3.95	3.76	0.454
Middle occipital gyrus	L	171	-36	-87	18	4.22	4.00	0.462
Parahippocampal gyrus	R	149	20	-22	-21	4.81	4.50	0.554
Hippocampal gyrus	R	148	11	-39	-6	3.88	3.70	0.558

Notes. BA, Brodmann area; L, left; R, right. Only clusters containing a minimum of 120 resampled voxels at an uncorrected $p < 0.001$ are reported. This combination of voxel-level and extent threshold is computed to be equivalent to a threshold of $p < 0.05$ after correction for multiple comparisons (see Methods). The rightmost column shows conventional application of family-wise error (FWE) corrected *p* values at the cluster level.
